# Two-year post-discharge costs of care among patients treated with transcatheter or surgical aortic valve replacement in Germany

**DOI:** 10.1186/s12913-017-2432-8

**Published:** 2017-07-11

**Authors:** Klaus Kaier, Frederike von Kampen, Hardy Baumbach, Constantin von zur Mühlen, Philip Hehn, Werner Vach, Manfred Zehender, Christoph Bode, Jochen Reinöhl

**Affiliations:** 1 Institute for Medical Biometry and Statistics, Faculty of Medicine and Medical Center – University of Freiburg, Freiburg im Breisgau, Germany; 2 Department of Cardiology, Heart Center Freiburg University, Freiburg im Breisgau, Germany; 3 Department of Cardiovascular Surgery, Robert-Bosch-Krankenhaus, Stuttgart, Germany; 4 Clinical Epidemiology, Center for Medical Biometry and Medical Informatics, Medical Center – University of Freiburg, Stefan-Meier-Str. 26, D-79104 Freiburg, Germany

**Keywords:** SAVR, TAVR, TAVI, Standardized unit costs, Two-part model, Patient-level data, I10, C24

## Abstract

**Background:**

This study presents data on post-discharge costs of care among patients treated with transcatheter or surgical aortic valve replacement over a two year period.

**Methods:**

Based on a prospective clinical trial, post-discharge utilization of health services and status of assistance were collected for 151 elderly patients via 2250 monthly telephone interviews, valued using standardized unit costs and analysed using two-part regression models.

**Results:**

At month 1 post-discharge, total costs of care are substantially elevated (monthly mean: €3506.7) and then remain relatively stable over the following 23 months (monthly mean: €622.3). As expected, the majority of these costs are related to in-hospital care (**~**98% in month 1 post-discharge and **~**72% in months 2–24). Patients that died during follow-up were associated with substantially higher cost estimates of in-hospital care than those surviving the two-year study period, while patients’ age and other patient characteristics were of minor relevance. Estimated costs of outpatient care are lower at month 1 than during the rest of the study period, and not affected by the event of death during follow-up. The estimated costs of nursing care are, in contrast, much higher in year 2 than in year 1 and differ substantially by gender and type of procedure as well as by patients’ age. Overall, these monthly cost estimates add up to €10,352 for the first and €7467.6 for the second year post-discharge.

**Conclusions:**

Substantial cost increases at month 1 post-discharge and in case of death during follow-up are the main findings of the study, which should be taken into account in future economic evaluations on the topic. Application of standardized unit costs in combination with monthly patient interviews allows for a far more precise estimate of the variability in post-discharge health service utilization in this group of patients than the ones given in previous studies.

**Trial registration:**

German Clinical Trial Register Nr. DRKS00000797.

**Electronic supplementary material:**

The online version of this article (doi:10.1186/s12913-017-2432-8) contains supplementary material, which is available to authorized users.

## Background

Any economic evaluation in healthcare heavily relies on estimates regarding the health consequences associated with different treatment approaches. When it comes to estimating long-run resource utilization, it is not uncommon to calculate these values using readily available event probabilities and average unit costs that are assumed to apply uniformly to all patients. For elderly patients with multiple severe co-morbidities, however, it is useful to more granularly estimate the costs of care for patients with different diseases, and to study in greater detail how different types of costs vary both over time and for various patient and treatment characteristics of interest [1].

The prevalence of acquired aortic valve stenosis is on the rise in the aging populations of the developed countries [2, 3]. The choice of treatment for these patients, however, remains controversial [4, 5]. The gold standard in the treatment of aortic valve stenosis has long been surgical aortic valve replacement (SAVR) [6, 7]. Since 2007, however, transcatheter aortic valve replacement (TAVR) has become established as a new standard of care for inoperable and high-risk patients [8, 9]. Ever since, clinical complications and long-term outcomes of TAVR have been the subject of extensive clinical research [10–12].

There is a considerable basket of studies that report TAVR and/or SAVR-related costs during the initial episode of hospitalization [13–23], or estimate the post discharge costs of care using event probabilities and average unit costs from the existing literature [24–31], and/or adapted results from the landmark Placement of Aortic Transcatheter Valve trial (PARTNER) [20, 21, 29], which however focused exclusively on the US healthcare system. In addition, the length of follow-up of the existing studies is short, and existing long-run follow-up costs are estimates-based data collected from various sources.

The aim of the present study was to close this gap of knowledge by evaluating two-year costs of care among high-risk patients with severe symptomatic aortic valve stenosis. In detail, comprehensive monthly cost measurements were conducted over a two year period a part of the prospective, medical-economic TAVI Calculation of Costs Trial (TCCT).

## Methods

### Data collection

The TAVI Calculation of Costs Trial (TCCT) was designed as a prospective observational multicenter cohort study on elderly patients with symptomatic AS receiving either SAVR, TAVR, or best medical therapy (DRUG) [32].

This study is part of TCCT and was approved by the institutional ethics committee (Research Ethics Committee Albert-Ludwigs-Universität Freiburg, Germany ID: 52/11), and registered in the German Clinical Trial Register (ID: DRKS00000797). Over a two-year period, a total of 2250 monthly telephone interviews were conducted by study nurse with 151 elderly patients treated with either TAVR (*n* = 85) or SAVR (*n* = 66). All patients referred to our centers between April 2011 and October 2013 were considered for inclusion into the study. Age above 75 years was deliberately chosen as an inclusion criterion. All treatment decisions were made by a study-independent “heart team” of cardiac surgeons and cardiologists according to best clinical practice [32].

In addition, a total of 70 follow-up interviews were conducted with 6 patients receiving drug-based therapy (DRUG, *N* = 6). Due to a lack of comparability, however, all results of DRUG patients were excluded from the main manuscript, but added to the online appendix (see Additional file 1: Table S1 and Additional file 2: Table S2).

### Calculating costs of care at the patient level

We applied a micro costing approach on the patient level. In detail, monthly records of medical resource utilization were used to calculate different costs of care for each patient-month separately. For visits at primary care physicians (€20.06) as well as medical emergency service or specialist consultants (€65.44), standardized unit costs from Bock et al. [2014] [33] were applied, representing costs as of 2011. For the small numbers of rescue services interventions, emergency admissions and outpatient hospital visits, however, no standardized unit costs were available and these consultations were therefore valued (at €222, €126 and €132, respectively) according to average expenditures or case specific reimbursement, as available. Episodes of hospitalization were valued according to documented hours of hospitalization in intensive care units (€1337.72 per 24 h) or general wards (€593.04 per 24 h), as suggested by Bock et al. [2014] [33]. The patients’ current status of assistance was valued according to the self-reported status of assistance and the documented care level in accordance with Bock et al. [2014] [33] as follows: Outpatient allowance for nursing care (€225, €430 and €685 for care level I, II and III), outpatient benefits in kind for nursing care (€440, €1040 and €1510 for care level I, II and III), inpatient short term care (€1923.61, €2340.29 and €2760.62 for care level I, II and III), inpatient long term care (€1782.38, €2224.93 and €2692.14 for care level I, II and III). Despite some minor inconsistencies in valuing every documented record of resource utilization, prices reflect 2011 values. Overall, four cost figures (*costs of nursing care*, *costs of outpatient care*, *costs of in-hospital care* and *total costs of care*) were distinguished, aggregated for each month and patient and used for analysis. Please note that the sum of the different cost figures, the *total costs of care*, does not include any type of indirect medical costs and therefore represents the perspective of all health care payers (health insurances and sickness funds), only.

### Statistical analysis

Skewed data is the main issue in statistical models in healthcare costs [34–36]. Beside the fact that the four cost figures were positively skewed, they were equal to zero during a considerable number of months because patients were not reliant on nursing care, did not see a physician and/or were not hospitalized in a given month. In order to accommodate these characteristics of the data, a two-part model approach was chosen for the regression analyses [37–39]. In two part models, a binary choice model is estimated for the probability of observing a zero versus positive outcome. Then, conditional on a positive outcome, an appropriate regression model is estimated for the positive outcome [40]. For part one of the applied models a logistic regression analysis was chosen to predict whether or not patients would utilize resources related to the respective costs figure. As recommended [36, 39, 41–44], a generalised linear model (GLM) with the log link and gamma distribution was chosen for the second part. In addition, the cluster option was used to address the fact that multiple monthly cost estimates are included in the dataset for the same patient. Marginal means from the combined models are shown on the raw scale (€ per month). This two-step procedure is used to first show the development of the monthly cost estimates over the two-year period by including time (in months) as a categorical covariate. Next, interaction terms between two covariates are included in order to separate the development of the monthly cost estimates across different patient groups. All analyses were performed using Stata 14 (Stata Corp., Texas. USA).

## Results

Table 1 provides an overview of baseline patient characteristics. For the 2250 documented patient months, a total of 2299 medical visits were recorded, including 216 cases of temporal hospitalization. As expected, temporal hospitalization was associated with substantial costs. A total of 27 of the 151 patients died during follow up.

**Table 1:** Patient characteristics and monthly parameters during two-year follow up

Baseline characteristics of 151 patients			
Age	82.06 ± 4.96		
Logistic EuroSCORE	15.14 ± 9.91		
Female	58.3%		
Patients aged 75–79 years	44.4%		
Patients aged 80–84 years	29.8%		
Patients aged > = 85 years	25.8%		
TAVR	56.3%		
AVR	43.7%		
Mean nursing care utilization during two-year follow up (*N* = 2250 patient months)			
No documentation of nursing care	82.7%		
Allowance for nursing care (outpatient)	5.1%		
Care benefits in kind (outpatient)	8.6%		
Short term care (inpatient)	0.2%		
Long term care (inpatient)	3.4%		
Number of medical visits during two-year follow up (*N* = 2250 patient months)			
Primary care physician	1568	€ 20.06	Bock et al. [2014]
Medical emergency service	13	€ 65.44	Bock et al. [2014]
Specialist consultant	368	€ 65.44	Bock et al. [2014]
Rescue service	26	€ 222	case specific reimbursement
emergency admission	5	€ 126	case specific reimbursement
outpatient hospital vist	103	€ 132	case specific reimbursement
Temporal hospitalization	216	€1337.72 (ICU)	Bock et al. [2014]
		€593.04 (general ward)	Bock et al. [2014]
Mean monthly costs and mortality during two-year follow up (*N* = 2250 patient months)			
Total costs (mean sd)	801.8 €	3345.3 €	
Costs of in-hospital care (mean sd)	638.5 €	3323.9 €	
Costs of outpatient care (mean sd)	33.9 €	47.3 €	
Costs of nursing care (mean sd)	129.4 €	387.4 €	
Mortality during follow-up	17.9%		

**Fig. 1:**
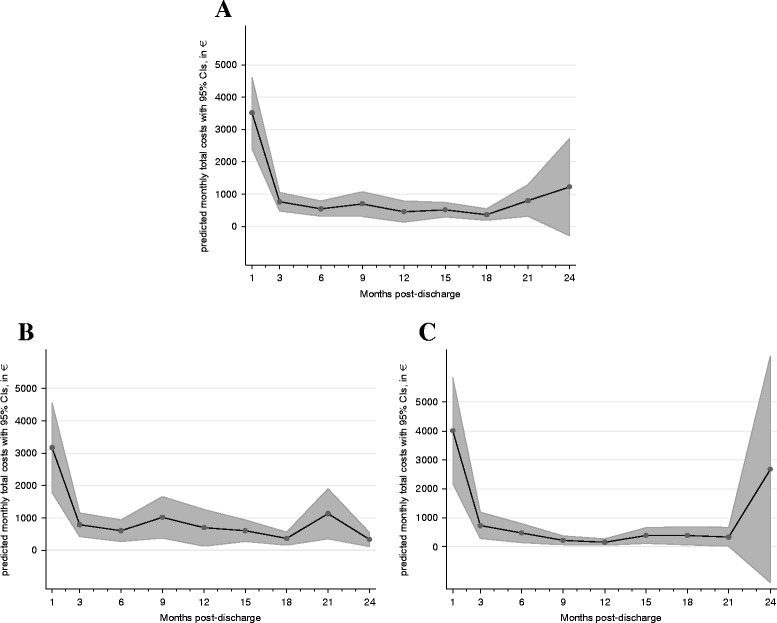
Two year monthly total costs of care estimates for patients treated for aortic valve stenosis. **a** All Patients. **b** TAVR Patients. **c** AVR Patients

Figure 1 provides monthly cost estimates regarding the four cost figures over the two-year period. At month 1 post-discharge, costs of in-hospital care are substantially increased, reaching €3439.1 (95%CI €2338.1–€4540.1), compared to only €452.6 (CI €316.9–€588.3) during the other months in the study period. In contrast, costs of outpatient care and costs of nursing care are lower for month 1 post-discharge, presumably due to the fact that many patients were discharged to another hospital directly.

**Fig. 2:**
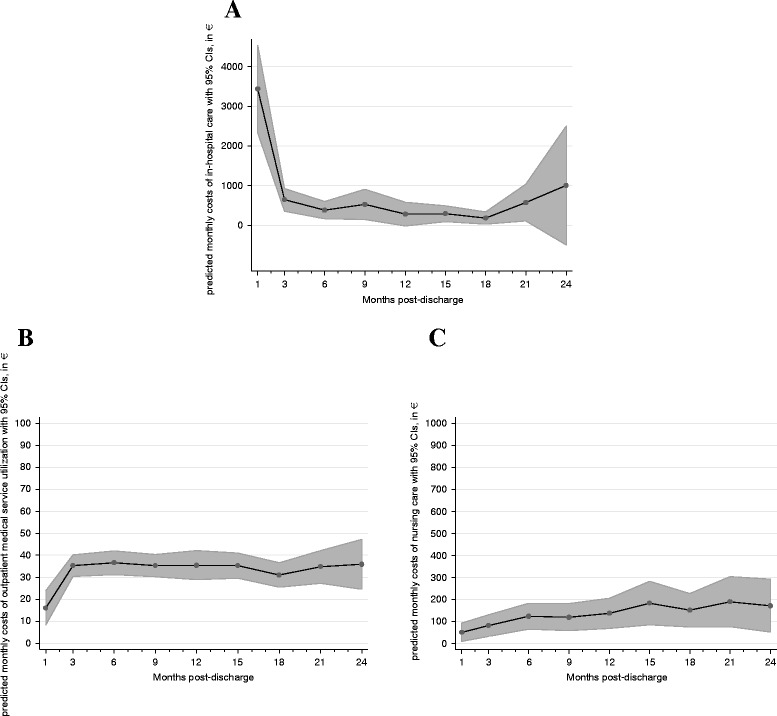
Two year monthly costs of in-hospital, outpatient and nursing of care estimates for patients treated for aortic valve stenosis. **a** Costs of in-hospital care. **b** Costs of outpatient care. **c** Costs of nursing care

Table 2 provides detailed estimates for costs of nursing care, costs of outpatient care, costs of in-hospital care and total costs of care and their value at month 1 post-discharge as well as during the other months in the study period. The costs of nursing care were separated into year 1 and year 2 figures due to their temporal development shown in Fig. 2.

**Table 2:** Two year monthly cost estimates of patients treated for aortic valve stenosis

	Total costs of care		Costs of in-hospital care		Costs of outpatient care		Costs of nursing care	
	Month 1	Month 2–24	Month 1	Month 2–24	Month 1	Month 2–24	Month 1–12	Month 13–24
Overall mean	3506.7	622.3	3439.1	452.6	16.12	35.13	104.7	174.0
	[2402.1,4611.4]	[473.6771.1]	[2338.1,4540.1]	[316.9588.3]	[8.377,23.85]	[32.40,37.86]	[55.65,153.8]	[89.25,258.7]
Male	3272.6	572.8	3311.0	441.0	17.60	37.33	67.99	113.7
	[1806.8,4738.4]	[333.4812.1]	[1902.4,4719.5]	[234.3647.6]	[8.822,26.39]	[33.17,41.50]	[10.04,125.9]	[21.24,206.2]
Female	3660.0	658.9	3512.9	461.9	14.74	33.53	133.5	215.5
	[2296.0,5024.0]	[476.9840.9]	[2201.9,4824.0]	[306.8617.0]	[7.418,22.07]	[30.00,37.05]	[62.05,204.9]	[100.1330.9]
TAVR	4247.5	711.0	3637.8	481.2	16.19	34.66	141.2	227.4
	[2472.9,6022.0]	[524.4897.6]	[2238.7,5036.9]	[314.3648.0]	[8.244,24.13]	[31.16,38.16]	[65.36,217.1]	[110.0,344.9]
AVR	2863.6	495.7	3182.2	411.3	16.19	35.78	56.03	94.47
	[1640.3,4086.8]	[264.9726.6]	[1854.2,4510.1]	[220.3602.2]	[7.963,24.42]	[31.53,40.04]	[6.171,105.9]	[13.84,175.1]
Patients aged 75–79 years	2779.6	509.9	3250.9	407.7	15.85	35.28	28.92	46.92
	[1453.1,4106.0]	[289.5730.3]	[1845.1,4656.7]	[223.5591.9]	[7.580,24.12]	[31.27,39.30]	[3.520,54.32]	[7.774,86.06]
Patients aged 80–84 years	4292.5	769.8	4297.0	560.3	15.47	35.77	114.5	173.3
	[2073.1,6511.9]	[461.5,1078.1]	[2221.7,6372.4]	[298.7821.8]	[7.464,23.47]	[30.68,40.86]	[29.22,199.8]	[58.11,288.5]
Patients aged ≥85 years	3840.7	629.6	3054.6	385.8	17.03	34.17	231.1	336.4
	[2313.3,5368.2]	[416.9842.3]	[1741.7,4367.4]	[212.7559.0]	[8.065,26.00]	[29.08,39.27]	[88.87,373.4]	[128.8544.0]
No death during follow-up	3013.4	500.7	2735.9	351.0	15.95	34.45	99.50	171.2
	[2013.5,4013.2]	[368.6632.8]	[1773.0,3698.8]	[235.9466.1]	[8.262,23.64]	[31.70,37.21]	[48.88,150.1]	[84.85,257.6]
Death during follow-up	9154.3	1703.2	8134.6	1285.9	15.93	41.49	137.8	232.8
	[3610.5,14,698.1]	[948.2,2458.2]	[4194.4,12,074.8]	[641.6,1930.2]	[5.420,26.43]	[31.56,51.42]	[−14.83,290.4]	[−9.125,474.7]
*N*	2250		2250		2250		2250	

Separate two part models, with one (only time: month 1 vs. month 2–24, or month 1–12 vs month 13–24) or two (time and sex or procedure or ...) categorical covariates are conducted with a logistic regression analysis for part one and a generalized linear model with the log link and gamma distribution for the second part. Marginal means for the combined models are shown on the raw scale (€ per month). 95% confidence intervals in brackets. All estimates reflect cost estimates in Euro (basis year 2011) from a societal perspective

Calculating the one-year total cost estimates from the predictions shown in Table 2 returns a mean of €10,352 for the first and €7467.6 for the second year post-discharge. As expected, the majority of these costs are related to in-hospital care, and the related cost estimates are thus also higher in year 1 (€8417.7) than in year 2 (€5431.2). In contrast the costs of outpatient care (year 1: €402.6; year 2: €421.6) and nursing care (year 1: €1256.4; year 2: 2088) are higher in the second year post-discharge. Cost estimates are broken down by gender, type of procedure, patients’ age and death during follow-up.

Patients that died during follow-up were associated with substantially higher month 1 and month 2–24 cost estimates of in-hospital care than those surviving the two-year study period (see Table 2). As a result, one-year total cost estimates for patients that died during the study period (year 1: €27,889.5; year 2: €20,438.4) are particularly higher than those for patients surviving the two-year follow-up (year 1: €8521.1; year 2: €6008.4). Interestingly, this relationship is also true with respect to the costs of in-hospital and nursing care, but not for the costs of outpatient care.

Estimated costs of outpatient care are lower at month 1 than during the rest of the study period, but relatively identical for males and females, the different types of procedure, patients’ age and even for the event of death during follow-up (See Table 2). The estimated costs of nursing care are, in contrast, much higher in year 2 than in year one and differ substantially by gender and type of procedure as well as by patients’ age (see Table 2). Please note that marginal effects for the first (probabilities of utilization) and second part (mean costs in case of utilization) of the two-part models are shown separately in Additional file 1: Table S1. With respect to the comparison of TAVR and SAVR-patients, for instance, probabilities and costs of in-hospital and outpatient care utilization were nearly identical, while probabilities and costs of nursing care utilization were substantially higher for TAVR patients.

Differences between the treatment groups are likely to interfere with pre-procedural differences regarding the baseline condition of patients that were not randomly assigned to the different treatment options. Instead, treatment decisions were made by a study-independent “heart team” of cardiac surgeons and cardiologists, which leads to a systematic risk selection [32], but corresponds to current best clinical practice [45]. As a result of this risk selection process, TAVR patients are considerably older (84.3 vs 80.7 years) and of higher risk according to the logistic EuroSCORE (mean EuroSCORE values TAVR: 19.26, SAVR: 9.83).

## Discussion

The goal of the present study was to estimate two-year post-discharge costs of care for TAVR and SAVR-patients. A considerable body of literature is available on the costs of care for TAVR and/or SAVR-patients; however, there are some major limitations: Usually, the length of follow-up in the existing studies is short, and the longer-term follow-up cost estimates are often based on models built from data collected from various sources. Iannaccone and Marwick (2015), for instance, recently reviewed the literature on the cost effectiveness of TAVR and SAVR in recent studies (published between 2012 and 2014) and found a huge range for total follow-up costs for the procedures of $336–$52,529 and $217–$51,992, respectively [46].

Many of the published studies that include follow-up costs use data from the same sources, especially the landmark PARTNER trial [20, 21]. Based on the results of the PARTNER trial, 1-year follow-up cost figures are available for 6 patient groups: Patients considered inoperable were randomly assigned to the treatment options (1) TAVR (*N* = 179) or (2) no treatment (*N* = 179). Patients considered at high risk for surgery were randomly assigned to the treatment options (3) Transcatheter aortic valve replacement via the transfemoral route (TF-TAVR, *N* = 239) or (4) SAVR (*N* = 217), or, if not anatomically suitable for TAVR via the TF approach, were randomly assigned to the treatment options (5) Transcatheter aortic valve replacement via the transapical route (TA-TAVR, *N* = 101) or (6) SAVR (*N* = 90). Overall, patients in these six treatment groups were comparably old (mean age in years: 83.1, 83.2, 83.9, 84.8, 83.1, 83.4, respectively [21, 47]) and were considered to be at high risk according to the logistic European System for Cardiac Operative Risk Evaluation (EuroSCORE) risk score (mean EuroSCORE values: 26.4, 30.4 in groups (1) and (2) as well as ~29 in the other groups [47, 48]). In comparison, the patients analysed in the present study were of comparable age (mean age in years: all patients: 82.06, TAVR: 83.59, SAVR: 80.09), but of substantially lower risk according to the logistic EuroSCORE (mean EuroSCORE values: all patients: 15.14, TAVR: 19.26, SAVR: 9.83). Hence our estimates indicate substantially lower post-discharge costs. It remains an open question whether this is due to differences in health care systems or differences in data collection procedures.

Patient mortality was similar to the rate encountered in comparable studies [32, 49]. Most importantly, patients were not randomly assigned to the different treatment options. Instead, treatment decisions were made by a study-independent “heart team” of cardiac surgeons and cardiologists, which leads to a systematic risk selection [32], but corresponds to current best clinical practice [45].

Calculating one-year cost estimates from the predictions shown in Table 2 returns a mean of €10,352 for all patients; €12,068.5 for the TAVR group and €8316.3 for the SAVR group, in 2011 € prices. In contrast, one year post-discharge costs reported in the PARTNER trial are reported in 2010 US dollars and given as $29,289 (~€23,431 using OECD PPP estimates), $24,787 (~€19,296), $23,540 (~€18,832), $18,856 (~€15,085), $19,959 (~€15,967) for the above described treatment options (1), (3), (4), (5) and (6), respectively [20, 21].

Collection of patient-level cost data during follow-up is a resource intensive exercise. Since memory is often impaired in elderly patients, it is essential that intervals between telephone interviews are kept as short as possible in order to collect reliable data. Application of standardized unit costs to the data collected in this manner allows for a far more precise estimate of the variability in post-discharge health service resource utilization in this group of patients than the ones given in previous studies.

Please note that there are a number of limitations: First of all, between-group differences in cost measurements should not, or at least only in part, be interpreted as treatment effects, as the different treatment groups are not randomly assigned but subject to a risk-driven patient selection. Secondly, there are substantial decreases in the number of cost measurements over the two year period and we may not assure whether dropouts were entirely noninformative. Finally, the applied temporal categorizations (month 1 vs month 2–24 for in-hospital and outpatient costs) imply the assumption of equal monthly costs between months 2–24.

Despite all these limitations, our results show that it is of major importance to consider country-specific cost estimates for further analysis rather than to solely rely on international evidence such as the PARTNER trial.

## Conclusions

Two year post-discharge costs of care are substantially impaired at month 1 post-discharge and in case of death during follow-up. In addition, application of standardized unit costs in combination with monthly patient interviews allows for a far more precise estimate of the variability in post-discharge health service utilization in this group of patients than the ones given in previous studies.

## Additional files

Additional file 1: Table S1. Marginal effects for the two parts of the two-part model separately. Marginal effects are shown for the two parts of the two-part model separately. As shown in Table 2, two-part models with one (time: month 1 vs. month 2–24, or month 1–12 vs month 13–24) or two (time and sex or procedure or ...) categorical covariates are conducted with a logistic regression analysis for part one and a generalised linear model with the log link and gamma distribution for the second part. Marginal effects for the combined models are shown on the raw scale (€ per month). 95% confidence intervals in brackets. All estimated prices reflect cost estimates in Euro (basis year 2011) from a societal perspective. (DOCX 38 kb)

Additional file 2: Table S2. Two year monthly cost estimates of patients treated for aortic valve stenosis (including also patients receiving drug-based therapy). Separate two-part models, with one (only time: month 1 vs. month 2–24, or month 1–12 vs month 13–24) or two (time and sex or procedure or ...) categorical covariates are conducted with a logistic regression analysis for part one and a generalised linear model with the log link and gamma distribution for the second part. Marginal effects for the combined models are shown on the raw scale (€ per month). 95% confidence intervals in brackets. All estimates reflect cost estimates in Euro (basis year 2011) from a societal perspective. (DOCX 25 kb)

## Abbreviations

AS: aortic stenosis
DRUG: control group of the TAVI Calculation of Costs Trial (TCCT) receiving best medical therapy
EuroSCORE: European System for Cardiac Operative Risk Evaluation
GLM: Generalised linear model
PARTNER trial: Placement of Aortic Transcatheter Valve trial
SAVR: Surgical aortic valve replacement
TA-TAVR: Transapical aortic valve replacement
TAVR: Transcatheter aortic valve replacement
TCCT: TAVI Calculation of Costs Trial
TF-TAVR: Transfemoral aortic valve replacement
