# Supplementary material for: Two-year post-discharge costs of care among patients treated with transcatheter or surgical aortic valve replacement in Germany
Source: BMC Health Serv Res. 2017 Jul 11;17:473. doi: 10.1186/s12913-017-2432-8 (PMC5504607; doi:10.1186/s12913-017-2432-8)
Supplement: Supplementary file 1 — Marginal effects for the two parts of the two-part model separately. Marginal effects are shown for the two parts of the two-part model separately. As shown in Table 2, two-part models with one (time: month 1 vs. month 2–24, or month 1–12 vs month 13–24) or two (time and sex or procedure or ...) categorical covariates are conducted with a logistic regression analysis for part one and a generalised linear model with the log link and gamma distribution for the second part. Marginal effects for the combined models are shown on the raw scale (€ per month). 95% confidence intervals in brackets. All estimated prices reflect cost estimates in Euro (basis year 2011) from a societal perspective. (DOCX 38 kb) [file 12913_2017_2432_MOESM1_ESM.docx]

# Two-year post-discharge costs of care among patients treated with transcatheter or surgical aortic valve replacement in Germany – Supplementary Tables.

# The European Journal of Health Economics

Klaus Kaier PhD^1,2^, Frederike von Kampen MD^2^, Hardy Baumbach MD^3^, Constantin von zur Mühlen MD^2^, Philip Hehn^1^, Werner Vach PhD^1^, Manfred Zehender MD PhD^2^, Christoph Bode MD^2^, Jochen Reinöhl MD^2^

^1^Clinical Epidemiology, Center for Medical Biometry and Medical Informatics, Medical Center – University of Freiburg, Germany

^2^Department of Cardiology, Heart Center Freiburg University, Germany

^3^Department of Cardiovascular Surgery, Robert-Bosch-Krankenhaus, Stuttgart, Germany

**Corresponding author:**

Klaus Kaier, Clinical Epidemiology, Center for Medical Biometry and Medical Informatics, Medical Center – University of Freiburg, Stefan-Meier-Str. 26, D-79104 Freiburg, Germany, Phone: (+49) 761 - 203 6807, Fax: ++49 (0)761 203-6711, email: kaier@imbi.uni-freiburg.de

**Table S1: Monthly probabilities and cost estimates from the two-part model**

|  |  | **Total costs of care** | |  | **Costs of in-hospital care** | |  | **Costs of outpatient care** | |  |  | **Costs of nursing care** | | |
| --- | --- | --- | --- | --- | --- | --- | --- | --- | --- | --- | --- | --- | --- | --- |
|  |  | **Part 1:**  **Probabilties**  **(of utilization)** | **Part 2:**  **Mean costs**  **(in case of utilization)** |  | **Part 1:**  **Probabilties**  **(of utilization)** | **Part 2:**  **Mean costs**  **(in case of utilization)** |  | **Part 1:**  **Probabilties**  **(of utilization)** | **Part 2:**  **Mean costs**  **(in case of utilization)** |  |  | **Part 1:**  **Probabilties**  **(of utilization)** | | **Part 2:**  **Mean costs**  **(in case of utilization)** |
| **Overall mean** | Month 1 | 51.40% | 6,818.7 € |  | 26.40% | 13,012.9 € |  | 25.00% | 64.5 € |  | Month 1-12 | 14.30% | 732.4 € | |
|  | Month 2-24 | 82.70% | 752.9 € |  | 5.55% | 8,162.8 € |  | 79.00% | 44.5 € |  | Month 13-24 | 22.80% | 762.4 € | |
|  |  |  |  |  |  |  |  |  |  |  |  |  |  | |
| **Male** | Month 1 | 52.80% | 6,203.8 € |  | 27.10% | 12,208.4 € |  | 26.40% | 66.6 € |  | Month 1-12 | 11.40% | 596.5 € | |
|  | Month 2-24 | 83.40% | 686.8 € |  | 5.73% | 7,697.5 € |  | 80.20% | 46.6 € |  | Month 13-24 | 18.70% | 607.8 € | |
|  |  |  |  |  |  |  |  |  |  |  |  |  |  | |
| **Female** | Month 1 | 50.50% | 7,248.6 € |  | 25.90% | 13,542.9 € |  | 24.00% | 61.5 € |  | Month 1-12 | 16.40% | 814.8 € | |
|  | Month 2-24 | 82.10% | 802.5 € |  | 5.41% | 8,538.9 € |  | 78.10% | 43.0 € |  | Month 13-24 | 26.00% | 830.3 € | |
|  |  |  |  |  |  |  |  |  |  |  |  |  |  | |
| **TAVR** | Month 1 | 52.50% | 8,090.8 € |  | 27.30% | 13,328.4 € |  | 25.40% | 63.6 € |  | Month 1-12 | 17.00% | 829.5 € | |
|  | Month 2-24 | 83.30% | 853.6 € |  | 5.79% | 8,313.6 € |  | 79.40% | 43.7 € |  | Month 13-24 | 26.70% | 850.6 € | |
|  |  |  |  |  |  |  |  |  |  |  |  |  |  | |
| **AVR** | Month 1 | 49.80% | 5,747.2 € |  | 25.10% | 12,662.4 € |  | 24.30% | 66.5 € |  | Month 1-12 | 10.50% | 533.4 € | |
|  | Month 2-24 | 81.80% | 606.3 € |  | 5.21% | 7,898.1 € |  | 78.40% | 45.7 € |  | Month 13-24 | 17.30% | 547.0 € | |
|  |  |  |  |  |  |  |  |  |  |  |  |  |  | |
| **Patients aged 75-79 years** | Month 1 | 48.40% | 5,737.4 € |  | 25.10% | 12,955.7 € |  | 24.40% | 64.9 € |  | Month 1-12 | 7.02% | 411.7 € | |
|  | Month 2-24 | 81.00% | 629.7 € |  | 5.20% | 7,847.7 € |  | 78.50% | 44.9 € |  | Month 13-24 | 11.70% | 401.7 € | |
|  |  |  |  |  |  |  |  |  |  |  |  |  |  | |
| **Patients aged 80-84 years** | Month 1 | 50.20% | 8,549.6 € |  | 28.70% | 14,974.9 € |  | 23.10% | 66.8 € |  | Month 1-12 | 17.10% | 669.0 € | |
|  | Month 2-24 | 82.00% | 938.4 € |  | 6.18% | 9,070.8 € |  | 77.30% | 46.3 € |  | Month 13-24 | 26.60% | 652.6 € | |
|  |  |  |  |  |  |  |  |  |  |  |  |  |  | |
| **Patients aged ≥85 years** | Month 1 | 57.60% | 6,669.2 € |  | 25.80% | 11,854.8 € |  | 28.20% | 60.5 € |  | Month 1-12 | 23.10% | 999.2 € | |
|  | Month 2-24 | 86.00% | 732.0 € |  | 5.37% | 7,180.8 € |  | 81.60% | 41.9 € |  | Month 13-24 | 34.50% | 974.7 € | |
|  |  |  |  |  |  |  |  |  |  |  |  |  |  | |
| **No death during** | Month 1 | 53.00% | 5,685.1 € |  | 23.10% | 11,824.7 € |  | 26.10% | 61.1 € |  | Month 1-12 | 14.00% | 713.0 € | |
| **follow-up** | Month 2-24 | 83.20% | 601.8 € |  | 4.96% | 7,079.9 € |  | 79.60% | 43.3 € |  | Month 13-24 | 22.60% | 756.3 € | |
|  |  |  |  |  |  |  |  |  |  |  |  |  |  | |
| **Death during follow-up** | Month 1 | 44.20% | 20,721.3 € |  | 41.60% | 19,569.1 € |  | 19.90% | 79.9 € |  | Month 1-12 | 16.50% | 833.7 € | |
|  | Month 2-24 | 77.60% | 2,193.5 € |  | 11.00% | 11,716.8 € |  | 73.30% | 56.6 € |  | Month 13-24 | 26.30% | 884.3 € | |
|  |  |  |  |  |  |  |  |  |  |  |  |  |  | |
| ***N*** |  | 2250 | 1,816 |  | 2250 | 154 |  | 2250 | 1701 |  |  | 2250 | 390 | |

Marginal means are shown for the two parts of the two-part model separately. As shown in Table 2, two-part models with one (time: month 1 vs. month 2-24, or month 1-12 vs month 13-24) or two (time and sex or procedure or ...) categorical covariates are conducted with a logistic regression analysis for part one and a generalized linear model with the log link and gamma distribution for the second part. 95% confidence intervals in brackets. All estimated prices reflect cost estimates in Euro (basis year 2011) from a societal perspective.

**Table S2: Two year monthly cost estimates of patients treated for aortic valve stenosis (including also patients receiving drug-based therapy)**

|  | **Total costs of care** | |  | **Costs of in-hospital care** | |  | **Costs of outpatient care** | |  | **Costs of nursing care** | |
| --- | --- | --- | --- | --- | --- | --- | --- | --- | --- | --- | --- |
|  | **Month 1** | **Month 2-24** |  | **Month 1** | **Month 2-24** |  | **Month 1** | **Month 2-24** |  | **Month 1-12** | **Month 13-24** |
| Overall mean | 3431.1 | 635.0 |  | 3358.7 | 457.1 |  | 16.91 | 35.11 |  | 111.0 | 185.2 |
|  | [2364.6,4497.6] | [489.7,780.4] |  | [2295.6,4421.8] | [324.9,589.4] |  | [9.322,24.49] | [32.40,37.82] |  | [62.64,159.3] | [101.4,269.0] |
|  |  |  |  |  |  |  |  |  |  |  |  |
| Male | 3207.6 | 582.9 |  | 3268.1 | 450.6 |  | 18.95 | 37.55 |  | 70.77 | 118.0 |
|  | [1824.5,4590.8] | [346.4,819.3] |  | [1914.4,4621.7] | [246.2,655.0] |  | [10.13,27.77] | [33.43,41.68] |  | [13.67,127.9] | [27.39,208.7] |
|  |  |  |  |  |  |  |  |  |  |  |  |
| Female | 3588.5 | 672.4 |  | 3411.8 | 462.3 |  | 15.26 | 33.37 |  | 142.3 | 228.8 |
|  | [2264.3,4912.7] | [496.1,848.7] |  | [2152.8,4670.8] | [312.5,612.1] |  | [8.159,22.36] | [29.87,36.87] |  | [72.28,212.4] | [115.4,342.2] |
|  |  |  |  |  |  |  |  |  |  |  |  |
| TAVR | 4079.3 | 711.8 |  | 3529.7 | 485.7 |  | 16.98 | 34.63 |  | 137.5 | 234.3 |
|  | [2388.7,5769.9] | [525.2,898.5] |  | [2173.6,4885.8] | [318.3,653.1] |  | [9.129,24.83] | [31.13,38.12] |  | [63.61,211.3] | [115.5,353.0] |
|  |  |  |  |  |  |  |  |  |  |  |  |
| AVR | 2756.5 | 496.9 |  | 3085.8 | 415.2 |  | 17.01 | 35.73 |  | 54.50 | 97.57 |
|  | [1582.3,3930.6] | [265.9,728.0] |  | [1791.7,4380.0] | [223.7,606.7] |  | [8.859,25.17] | [31.49,39.98] |  | [5.983,103.0] | [14.64,180.5] |
|  |  |  |  |  |  |  |  |  |  |  |  |
| DRUG | 6582.7 | 1002.1 |  | 3353.3 | 496.1 |  | 12.83 | 35.92 |  | 358.9 | 461.1 |
|  | [1848.3,11317.0] | [583.0,1421.2] |  | [1229.9,5476.7] | [159.3,833.0] |  | [1.670,24.00] | [18.70,53.13] |  | [106.3,611.4] | [175.9,746.4] |
|  |  |  |  |  |  |  |  |  |  |  |  |
| Patients aged 75-79 years | 2668.8 | 511.2 |  | 3179.9 | 413.9 |  | 16.93 | 35.21 |  | 29.11 | 47.87 |
|  | [1439.6,3898.0] | [290.8,731.5] |  | [1823.0,4536.8] | [229.4,598.3] |  | [8.680,25.19] | [31.21,39.22] |  | [4.135,54.09] | [8.744,86.99] |
|  |  |  |  |  |  |  |  |  |  |  |  |
| Patients aged 80-84 years | 4183.3 | 779.2 |  | 4193.1 | 567.8 |  | 16.88 | 36.14 |  | 116.7 | 178.2 |
|  | [2073.5,6293.1] | [476.1,1082.3] |  | [2209.3,6176.8] | [309.9,825.6] |  | [8.716,25.05] | [31.11,41.17] |  | [33.22,200.2] | [64.10,292.2] |
|  |  |  |  |  |  |  |  |  |  |  |  |
| Patients aged ≥85 years | 3827.8 | 659.1 |  | 2976.3 | 389.6 |  | 16.83 | 33.86 |  | 241.7 | 352.2 |
|  | [2338.0,5317.5] | [458.3,859.8] |  | [1740.9,4211.7] | [227.5,551.7] |  | [8.561,25.09] | [28.82,38.90] |  | [108.9,374.5] | [158.4,546.1] |
|  |  |  |  |  |  |  |  |  |  |  |  |
| No death during follow-up | 2939.1 | 516.2 |  | 2645.5 | 356.4 |  | 16.93 | 35.21 |  | 107.2 | 183.2 |
|  | [1971.0,3907.2] | [386.7,645.6] |  | [1721.5,3569.5] | [243.8,469.0] |  | [8.680,25.19] | [31.21,39.22] |  | [56.77,157.6] | [97.71,268.8] |
|  |  |  |  |  |  |  |  |  |  |  |  |
| Death during follow-up | 8408.8 | 1688.3 |  | 7785.6 | 1286.4 |  | 16.88 | 36.14 |  | 134.8 | 228.4 |
|  | [3417.9,13399.6] | [955.2,2421.4] |  | [4146.2,11425.1] | [669.1,1903.7] |  | [8.716,25.05] | [31.11,41.17] |  | [-9.111,278.7] | [-1.672,458.4] |
|  |  |  |  |  |  |  |  |  |  |  |  |
| *N* | 2320 | 2320 |  | 2320 | 2320 |  | 2320 | 2320 |  | 2320 | 2320 |

Separate two-part models, with one (only time: month 1 vs. month 2-24, or month 1-12 vs month 13-24) or two (time and sex or procedure or ...) categorical covariates are conducted with a logistic regression analysis for part one and a generalized linear model with the log link and gamma distribution for the second part. Marginal means for the combined models are shown on the raw scale (€ per month). 95% confidence intervals in brackets. All estimates reflect cost estimates in Euro (basis year 2011) from a societal perspective.
